# Image-Based Chemical Structure Determination

**DOI:** 10.1038/s41598-017-07041-x

**Published:** 2017-07-28

**Authors:** Johannes Ofner, Florian Brenner, Karin Wieland, Elisabeth Eitenberger, Johannes Kirschner, Christoph Eisenmenger-Sittner, Szilvia Török, Balazs Döme, Thomas Konegger, Anne Kasper-Giebl, Herbert Hutter, Gernot Friedbacher, Bernhard Lendl, Hans Lohninger

**Affiliations:** 1 Institute of Chemical Technologies and Analytics, TU Wien, Getreidemarkt 9, 1060 Vienna, Austria; 2 Institute of Solide State Physics, TU Wien, Wiedner Hauptstrasse 8, 1040 Vienna, Austria; 3 National Korányi Institute of Pulmonology, Budapest, Hungary; 4 Division of Thoracic Surgery, Department of Surgery, Comprehensive Cancer Center, Medical University of Vienna, Vienna, Austria; 5 Department of Thoracic Surgery, Semmelweis University and National Institute of Oncology, Budapest, Hungary; 6 Department of Biomedical Imaging and Image-guided Therapy, Division of Molecular and Gender Imaging, Medical University of Vienna, Vienna, Austria

## Abstract

Chemical imaging is a powerful tool for understanding the chemical composition and nature of heterogeneous samples. Recent developments in elemental, vibrational, and mass-spectrometric chemical imaging with high spatial resolution (50–200 nm) and reasonable timescale (a few hours) are capable of providing complementary chemical information about various samples. However, a single technique is insufficient to provide a comprehensive understanding of chemically complex materials. For bulk samples, the combination of different analytical methods and the application of statistical methods for extracting correlated information across different techniques is a well-established and powerful concept. However, combined multivariate analytics of chemical images obtained via different imaging techniques is still in its infancy, hampered by a lack of analytical methodologies for data fusion and analysis. This study demonstrates the application of multivariate statistics to chemical images taken from the same sample via various methods to assist in chemical structure determination.

## Introduction

Chemical imaging has become a workhorse in analytical chemistry due to advanced method development, improved imaging speed, lower detection limits, and increased computational power. All major analytical methods have been extended for chemical imaging purposes, resulting in improved understanding of the heterogeneity and complexity of samples of interest. Successful chemical structure determination (CSD) combines different complementary analytical techniques applied to the same sample to generate a comprehensive analytical understanding of the sample under investigation. While the combination of complementary analytical methods to solve scientific questions regarding complex samples or chemical systems is a fundamental working principle in analytical chemistry, the application of this approach in chemical imaging has been rarely reported.

‘Multimodal imaging,’ ‘correlative imaging,’ and ‘data fusion’ are common catchphrases that refer to the topic of combined image-based analysis. In these approaches, the data from each technique is analysed separately and the resulting images are combined. Correlative microscopy using light and electron microscopy is discussed in detail by Hayat^1^. Modern sample preparation techniques have been developed to assist this correlative approach^2, 3^. The advantages of image fusion of imaging mass spectrometry and microscopy are described by Van de Plas *et al*.^4^. Approaches toward data correlation to address biological questions are reported by Cunha *et al*.^5^, focusing on available software systems for multimodal imaging and giving the example of correlating secondary ion mass spectrometric (SIMS) images with those obtained by transmission electron microscopy. A similar approach is reported for energy dispersive X-ray (EDX) spectroscopy and electron microscopy^6^. Outside of the biological and life-science communities, data fusion became a topic of interest in surface science when time-of-flight-SIMS (ToF-SIMS) images were combined with scanning electron microscopic (SEM) images and analysed using multivariate statistics^7^ and when images from X-ray photoelectron spectroscopy and atomic force microscopy (AFM) were fused^8^. AFM has also been successfully fused with data obtained from laser scanning microscopy^9^. All these studies provide additional scientific output by combining a chemical imaging technique with high-resolution imaging (HRI) and/or topographic imaging techniques such as SEM or AFM. A different approach to correlative imaging, however, is the fusion of different modalities of the same fundamental physico-chemical measurement principle of imaging techniques, that is, by fusing near-infrared, mid-infrared, and Raman micro-spectroscopic (RMS) images with subsequent multivariate data analysis^10^. A similar multimodal approach was applied to imaging tissues using SIMS and matrix-assisted laser desorption/ionization (MALDI)^11^. Another correlative technique is reported for imaging chromosomes by cryo-fluorescence and soft X-ray tomography (SXT)^12^. By the application of SXT, a three-dimensional reconstruction of the correlated dataset could be achieved. Recently, correlative 3-dimensional micro-spectroscopy of a single catalyst particle has been reported^13^. The detailed methodical approach is of this technique is summarized in a recent review^14^.

Data fusion of single chemical images with HRI or topographic images or imaging of a sample by using different modalities of a single technique providing similar information, for instance, vibrational spectroscopy (infrared and RMS) or mass spectrometry (SIMS and MALDI), do not fulfil the requirements of CSD because hardly any complementary chemical information is generated. SIMS and RMS images have been correlated to visualize cell-scale molecular distributions^15^. Here, the complementary information of mass spectrometric and vibrational imaging are combined and compared at the image level. This is also reported for the combined use of laser-induced breakdown spectroscopy and laser ablation inductively coupled plasma mass spectrometry^16^. Contrary to these examples, fusion on the level of recorded datasets obtained from elemental (EDX) and vibrational (Raman) micro-spectroscopy (RMS) was reported in 2014^17^. The advantages of the combined multivariate analytical approach for this so-called multisensor hyperspectral imaging (MSHSI) dataset were recently compared to side-by-side interpretation of images^18^.

To generate an approach to image-based CSD that is both comprehensive and complementary, imaging data obtained via different spectroscopic techniques must first be combined and then jointly analysed. Therefore, the imaging techniques must be non-destructive, must exhibit comparable spatial resolutions, and must return complementary chemical information. Additionally, the sample area of interest must be accessible by all these techniques. Elemental, vibrational, and mass spectrometric imaging can fulfil these requirements using EDX imaging applied by an electron microscope (where SEM also supplies the HRI), RMS imaging, and ToF-SIMS imaging (in the following paragraphs referred to as SIMS). RMS and EDX imaging are both non-destructive techniques; thus, multisensor fusion of these datasets with a SIMS dataset is possible when SIMS imaging is applied as the last technique. With spatial resolutions down to 50 nm for EDX, down to 100 nm for SIMS, and down to 200 nm for RMS, these techniques are comparable.

**Figure 1:**
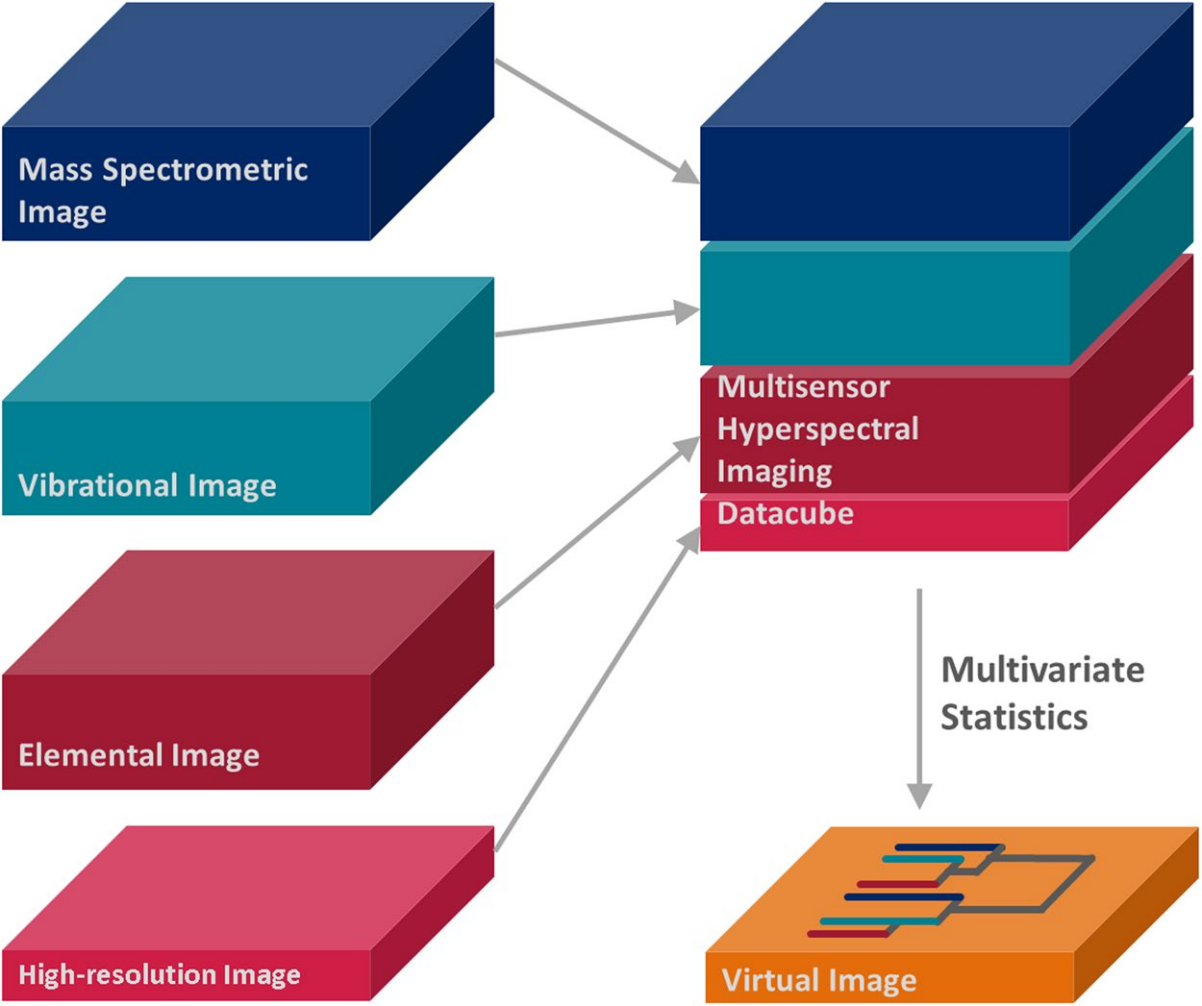
Basic concept of multisensor hyperspectral imaging with subsequent multivariate statistics to generate an analytical representation of a sample for image-based chemical structure determination.

The present study demonstrates image-based CSD by merging EDX, RMS, and SIMS imaging datasets into a combined MSHSI datacube; subsequently, multivariate statistics are applied (Fig. 1) to generate a comprehensive analytical representation of the image. Spectral descriptor (SPDC)^17^ based principal component analysis (PCA)^19^, k-means cluster analysis^20^ and hierarchical cluster analysis (HCA)^21^, as well as vertex component analysis (VCA)^22^, are applied to analyse the combined MSHSI datasets^18^. The application of multivariate MSHSI to image-based CSD will be introduced via the combined analysis of copper sulphide particles deposited on a purified aluminium substrate. Subsequently, with the aim of demonstrating the generic applicability of this new methodology to samples from the fields of life science, materials science, and geoscience, additional examples using, respectively, tumour cells, technical ceramics, and an environmental aerosol sample are given.

## Results

### Copper sulphide particles

To analyse the combined dataset obtained from RMS, EDX, and SIMS imaging in combination with the HRI of copper sulphide particles using MSHSI, spectral descriptors (SPDCs) were defined to extract the spectral information from the datacube. Copper sulphide is characterized by three Raman bands at 270 (ν(Cu–S)), 473 (ν(S–S)), and 917 (2ν(S–S)) cm^−1^ ^23–25^. Additionally, Kα X-ray emissions are expected in the EDX spectrum for aluminium (background), copper, and sulphur. In the SIMS datacube, mass-to-charge (*m*/*z*) 26.98 for the aluminium substrate as well as 62.93 and 64.93 for the two copper isotopes are significant for the chosen system. Because the ToF-SIMS was operated in positive-ion mode, no SIMS signal of sulphur is expected.

**Figure 2:**
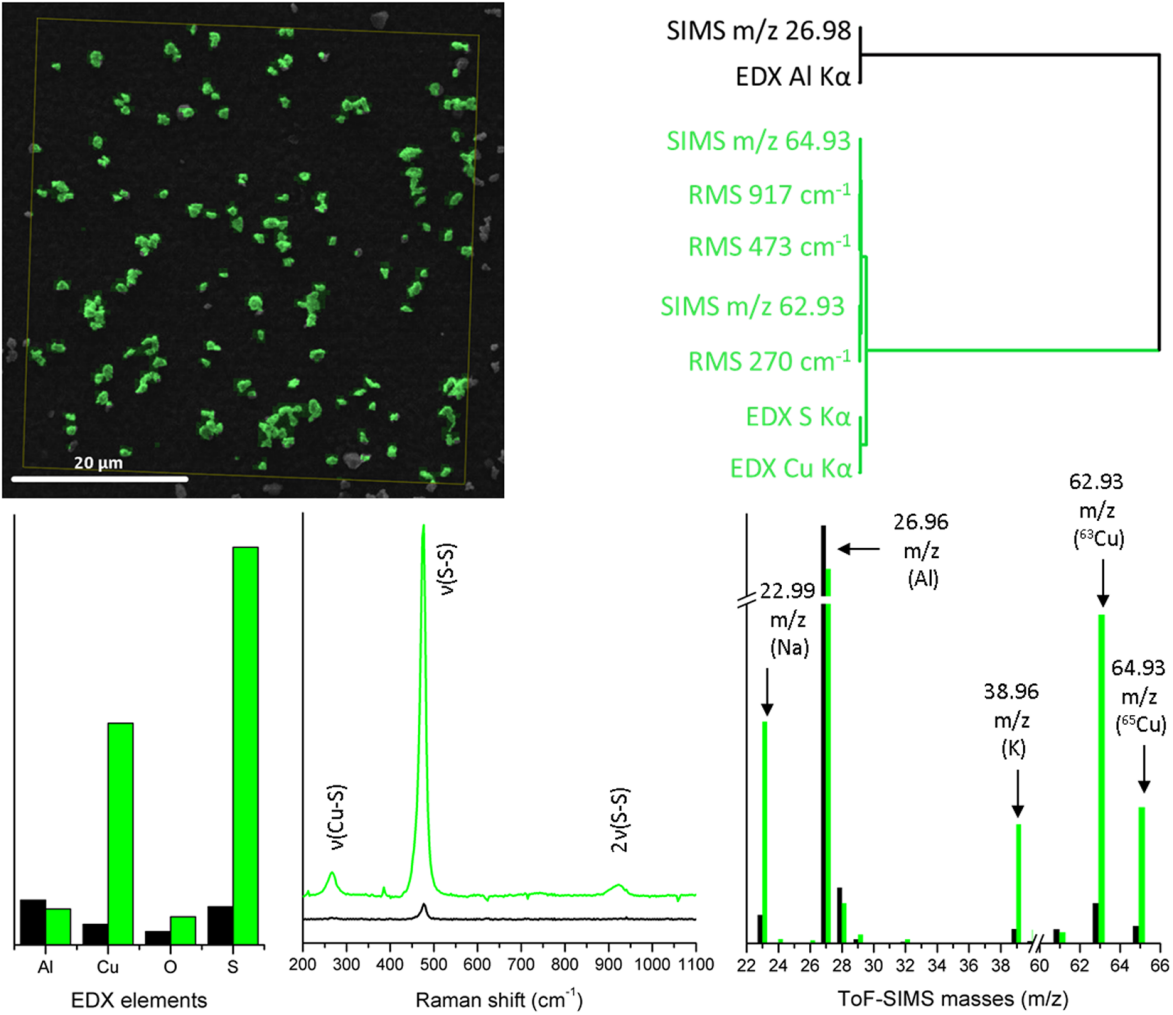
Superposition of the high-resolution SEM image with the CuS particle cluster of the HCA of the loadings of the PCA, dendrogram of the PCA-HCA indicating the background (black) and CuS particle cluster (green), and the extracted cluster spectra from the k-means clustering.

The multisensor hyperspectral dataset was analysed using PCA on the basis of the defined SPDCs (more details are given in the Methods section). The first principal component describes 65.77% of the overall dataset variance. Based on the PCA of standardized SPDCs, an HCA of the loadings of the first principal component was carried out (Fig. 2, upper part). A detailed description of all applied multivariate statistical methods can be found at Ofner *et al*.^18^. The colours in Fig. 2 and the following figures represent the different unravelled species. The colour-coding links the detected species in the SEM or optical images with the related dendrograms of the clustering algorithms and the extracted mean spectra. Due to the fact, that all demonstrated spectra origin from multivariate analysis (especially k-means clustering, HCA or VCA) and are standardized mean spectra, no meaningful units can be given for the intensities of the ordinates of the spectra. Therefore, labels and units are not shown.

The dendrogram of the HCA of the loadings of the PCA (Fig. 2, upper part) significantly differentiates the background cluster (SIMS *m*/*z* 26.98 of aluminium and the EDX X-ray Kα emission of aluminium) and the particle cluster. This cluster contains all three Raman bands of copper sulphide (917 cm^−1^, 473 cm^−1^, and 270 cm^−1^), the copper isotopes from the ToF-SIMS dataset (*m*/*z* 64.93 and *m*/*z* 62.93), and the related X-ray emissions from the EDX of the SEM (S Kα and Cu Kα).

The superposition of the SEM image and the sub-cluster image of the PCA-HCA of the MSHSI datacube indicates that not all particles are related to this sub-cluster of copper sulphide. Because the first principal component describes only 65.77% of the overall dataset, additional chemical information is assumed to be hidden in the MSHSI. A more detailed examination reveals the presence of oxygen (EDX), as well as impurities of Na (SIMS *m*/*z* 22.99), K (SIMS *m*/*z* 38.97), Ca (SIMS *m*/*z* 39.97), and other elements, originating from the sample preparation process. However, the major component of interest, copper sulphide, could be clearly identified within the first principal component.

To extract the combined component spectra, k-means clustering standardized SPDCs of the MSHSI datacube, choosing two expected clusters, was performed (Fig. 2, lower part). The extracted cluster spectra exhibit the expected features. The EDX spectrum reveals the presence of Kα emissions of copper and sulphur for the green cluster, in addition to a slight increase of oxygen, which indicates the presence of copper oxide. The extracted Raman spectrum exactly replicates the Raman spectrum of covellite (CuS)^26^. The SIMS spectrum identifies the two copper isotopes ^63^Cu and ^65^Cu at their atomic weights of 62.93 and 64.93 and their expected isotope ratios of 69.17% and 30.83%, respectively. Additionally, the SIMS spectrum of the CuS sub-cluster (Fig. 2, green cluster) exhibits impurities of sodium (22.99 *m*/*z*) and potassium (38.96 *m*/*z*). The substrate (Fig. 2, black cluster) is characterized by an EDX Kα emission of aluminium, a nearly flat Raman baseline with traces of the CuS Raman spectrum, which is caused by the multisensor fusion of the single datasets with varying spatial resolution of the methods, and higher abundance of the mass 26.96 *m*/*z* (aluminium) in the SIMS spectrum.

The application of MSHSI to the simple example of CuS particles shows the advantages of this technique. While EDX allows rudimentary identification of the component elements, RMS reveals the chemical bonding and allows attribution to CuS, while SIMS confirms the presence of copper by identifying the masses and ratios of the isotopes as well as providing additional information on minor constituents, impurities, and contaminants. All this sample-specific information, which is necessary for definite chemical structure determination, is represented within a single sub-cluster of the multivariate k-means clustering, which proves the linkage of the individual analytical methods.

### Tumour cells

**Figure 3:**
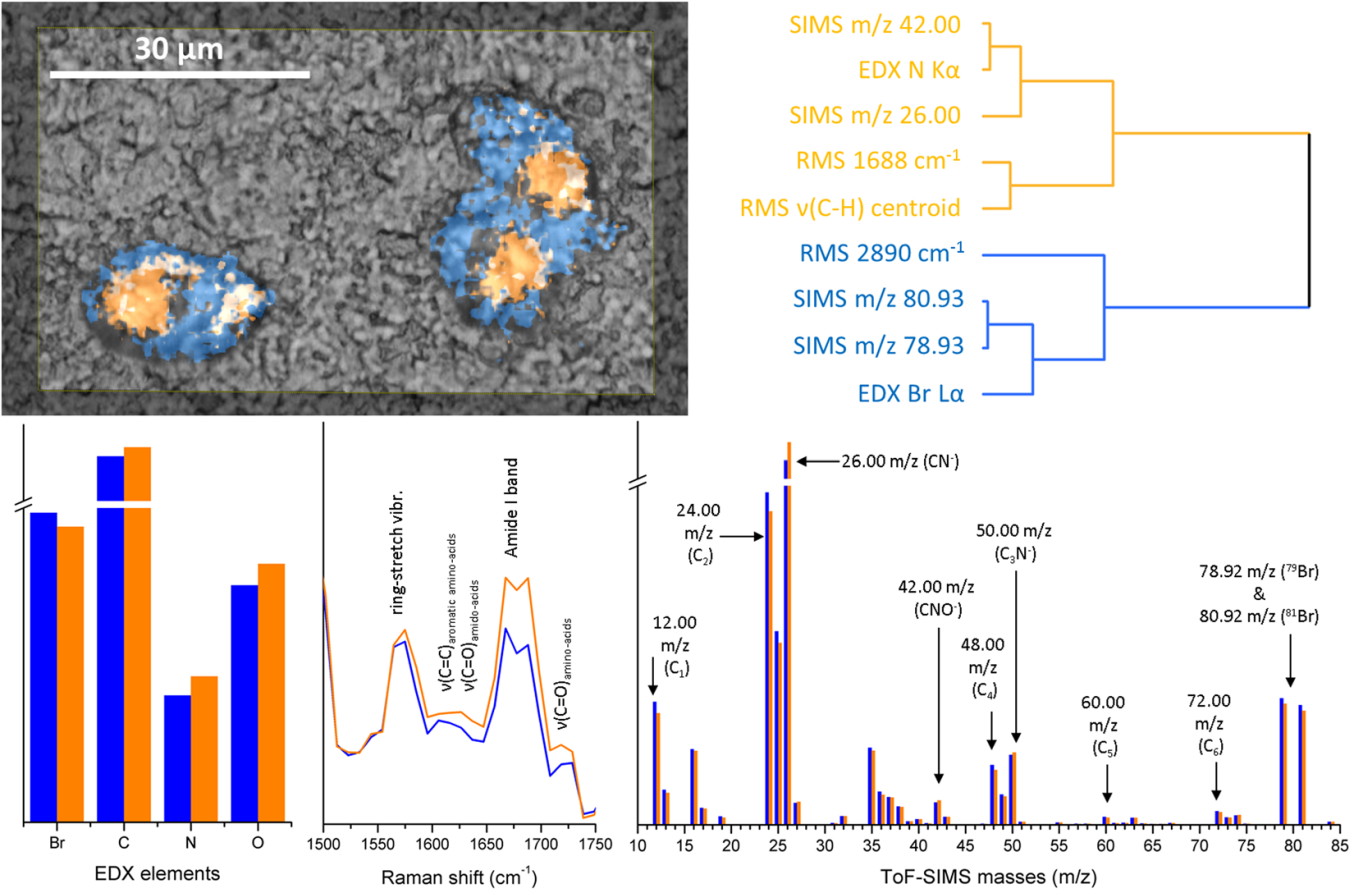
Superposition of a light-microscopic image of two tumour cells and results from the HCA of the PCA loadings and extracted cluster spectra from k-means clustering of the MSHSI dataset.

Tumour cells treated with a bromine-containing prodrug were also imaged using MSHSI (Fig. 3). To analyse the combined MSHSI datacube of this sample, HCA of the PCA loadings based on standardized SPDCs was performed, based on the concept of SPDCs (Fig. 3, upper part). The two sub-clusters of the HCA of the PCA show a distinction between the nucleus surrounded by the rough endoplasmic reticulum for protein biosynthesis (Fig. 3, orange sub-cluster) and the cytoplasm of the cell (Fig. 3, blue sub-cluster). The superposition of the cluster image with the optical image shows that both cells undergo cell division. The orange sub-cluster is defined by the SPDCs of the EDX nitrogen signal, the SIMS masses 26.00 and 42.00 *m*/*z*, which can be attributed to CN^−^ and CNO^−^ fragments, and the Raman amide I band at 1688 cm^−1^ as well as a centroid spectral descriptor, which is assigned to the ν(C–H) stretch region and exhibits a positive value at the location of the nucleus. The blue sub-cluster is characterized by a Raman band at 2890 cm^−1^, the ν(C–H) of –CH_2_– groups, which can be assigned to lipids, the EDX Lα emission of bromine, and the ToF-SIMS masses 78.93 *m*/*z* and 80.93 *m*/*z*, which represent the two bromine isotopes ^79^Br and ^81^Br, respectively.

The component spectra (Fig. 3, lower part) of the cell core and the cytoplasm were extracted using k-means clustering of standardized SPDCs. After q-normalization, the best results for the clustering process were obtained by choosing three expected clusters. The second k-means cluster was excluded from further processing because this cluster represents the margin of the cells, where a strong contribution from the background and background impurities is visible. The two remaining sub-clusters of the k-means clustering, which are comparable to the clusters of the HCA of the PCA loadings, improve the chemical interpretation of the nucleus and cytoplasm clusters. The nucleus sub-cluster (Fig. 3, orange spectra) exhibits an enhanced band at 1620 cm^−1^, which can be assigned to the ν(C = O) of amino acids^27^ or the ν(C = C) of aromatic amino acids^28^ as well as the common ν(C = O) of esters^28^ around 1730 cm^−1^. Further, a strong increase of the amide I band at 1680 cm^−1^ is visible^29^. Additionally, both clusters exhibit the ring-stretch vibration of purine bases at about 1575 cm^−1^ ^29^. The RMS spectra thus indicate an enhanced presence of proteins in the orange sub-cluster. This is also supported by the SIMS mass spectra, which show increased intensities in this sub-cluster of 26.00 (CN^−^), 42.00 (CNO^−^), and 50 (C_3_N^−^) *m*/*z*. In contrast, the blue sub-cluster of the HCA dendrogram of the PCA loadings depicting the cytoplasm supports the assignment of the Raman vibrations of lipids by the SIMS fragments 12 (C), 24 (C_2_), 48 (C_4_), 60 (C_5_), and 72 (C_6_) *m*/*z*, which represent lipid fragments. The increased presence of lipids in tumour cells, which is highlighted by the RMS centroid SPDC and the SIMS spectrum of the blue sub-cluster, is also reported in the literature^30^. The EDX spectrum indicates increased presence of carbon, oxygen, and nitrogen at the location of the cell cores, while the bromine-containing drug appears primarily in the cytoplasm. This distribution of bromine is also confirmed by the two SIMS masses 78.92 *m*/*z* (^79^Br) and 80.92 *m*/*z* (^81^Br). Thus, besides the localization of the cell cores, the distribution of the bromine-containing drug inside the cells could also be visualized using MSHSI.

### Ceramic composite

As an example of the application of image-based chemical structure determination to the field of materials science, a ceramic composite prepared by the polymer-precursor method was analysed. The synthesis of this technical ceramic, which consists of ZrO_2_-reinforced mullite (3Al_2_O_3_·2SiO_2_), was based on the thermal conversion of methyl-polysilsesquioxane to highly reactive SiO_2_, which subsequently reacted with Al_2_O_3_ to form a continuous mullite matrix. Additionally, ZrO_2_ (yttrium-stabilized) was incorporated as particulate reinforcement. The full synthesis procedure and SEM image-based analytics have been described in previous studies^31, 32^.

**Figure 4:**
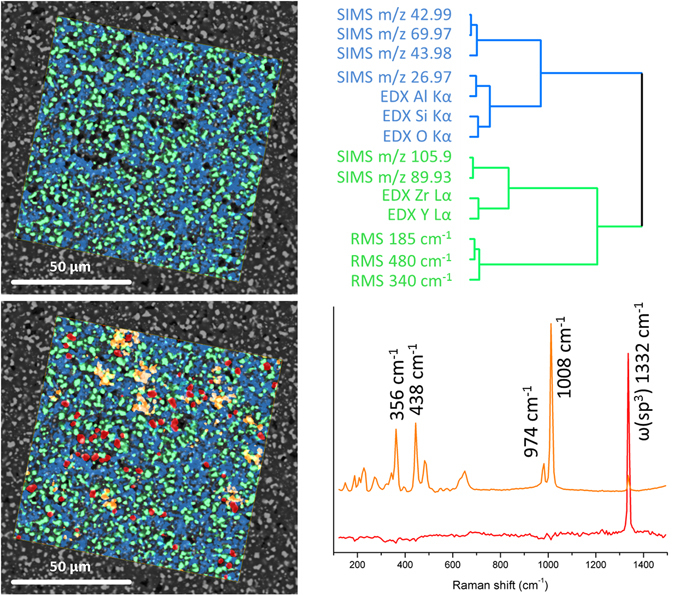
Superposition of the SEM image of the ceramic mullite/ZrO_2_ composite with the image of the HCA of the PCA loadings (upper part) and with the extracted endmembers of the VCA (lower part), which represents the diamond Raman spectrum (red) and the ZrSiO_4_ Raman spectrum (orange).

Based on the knowledge of the initial chemical composition, SPDCs were chosen to analyse the MSHSI dataset. HCA of the PCA loadings based on standardized SPDCs was performed, which indicated the main expected components (Fig. 4, upper part). The dendrogram is characterized by two main sub-clusters. The green sub-cluster in Fig. 4 represents the yttrium-stabilized zirconia. This sub-cluster contains the EDX Lα X-ray emissions of zirconium and yttrium. Furthermore, the corresponding SIMS masses 89.93 *m*/*z* of Zr and 105.9 *m*/*z* of ZrO are included. The three RMS bands at 185 (two bands at 180 and 192 cm^−1^), 340, and 480 cm^−1^ finally allow the assignment of this sub-cluster to monoclinic ZrO_2_^33^. Hence, the green sub-cluster represents the yttrium-stabilized ZrO_2_. The blue sub-cluster represents the matrix phase, in which the EDX X-ray Kα emissions of silicon, oxygen, and aluminium and the corresponding SIMS masses of 26.97 *m*/*z* (Al), 42.99 *m*/*z* (AlO), 43.98 *m*/*z* (SiO), and 69.97 *m*/*z* (Al_2_O) can be found. These SPDCs indicate that this sub-cluster is representative of mullite (3Al_2_O_3_·2SiO_2_). However, the Raman bands of these species are not accessible due to the presence of the strong ZrO_2_ bands.

The overall sample is characterized by pores, which are artefacts of the polishing process. Analysing these holes in detail revealed an enhanced amount of carbon (EDX Kα). Therefore, an additional species was expected. The EDX dataset only exhibited the information on the carbon content. Further, no significant masses could be found in the SIMS dataset for these parts of the sample. VCA was applied on the RMS portion of the MSHSI dataset and a spectral endmember, corresponding to the holes in the sample, was extracted (Fig. 4, lower part, red RMS spectrum). This endmember is characterized by a single Raman band at about 1332 cm^−1^ and correlated to high carbon content. Based on the literature, the red spots in Fig. 4 (lower part) can be assigned to diamond^34^. This vibration is evoked by the interpenetrating cubic sublattice and can be assigned to a lattice vibration of the sp^3^ carbon bonds (ω(sp^3^))^35^. Diamond paste was used to polish the sample, and the red spots indicate areas of diamond residue. The VCA algorithm also revealed an additional spectral endmember in the Raman datacube (Fig. 4, lower part, orange spectrum). According to the literature^36^ and the RRUFF database^26^, as well as expectations based on the synthesis method^31^, this VCA endmember can be attributed to ZrSiO_4_, which forms at the interface between the ZrO_2_ particles and the mullite matrix.

By applying PCA-HCA and VCA to the MSHSI dataset of the ceramic sample, four main chemical species could be extracted from the SEM image. The yttrium-stabilized ZrO_2_ is well represented by the RMS, EDX, and SIMS sub-datasets. By superimposition with the SEM image, the white particles could be identified as 3Y-ZrO_2_. The mullite matrix is congruent with the gray areas in the SEM image and is characterized by the EDX and SIMS sub-datasets of the MSHSI datacube. Additionally, the RMS sub-dataset revealed the presence of ZrSiO_4_ by PCA-HCA and diamond residues by VCA endmember extraction from the RMS sub-dataset, which was also confirmed by the enhanced carbon signal in the EDX dataset.

### Environmental aerosol

Atmospheric aerosols are complex mixtures of analytes from natural or anthropogenic sources that are formed and transformed in the environment. A combined analytical approach to this class of samples increases the value of the chemical analysis and enhances the understanding of the entire environmental system. During atmospheric sampling in the city of Vienna, a nearby building that had been affected by a fire was torn down.

The sample was imaged and the datasets were fused into the MSHSI datacube. Because this sample exhibited a high number of different chemical species, SPDCs had to be defined by an iterative process. Restrictive clustering algorithms such as k-means clustering would hamper the interpretation of the dataset because the expected number of clusters must be known in advance. An estimation of the expected number of species based on analysis of the eigenvalue plot of the PCA revealed eight to ten different chemical species. To allow a top-down analysis of these species, SPDC-based HCA (Ward’s method with Euclidean distances) of the overall dataset was performed.

**Figure 5:**
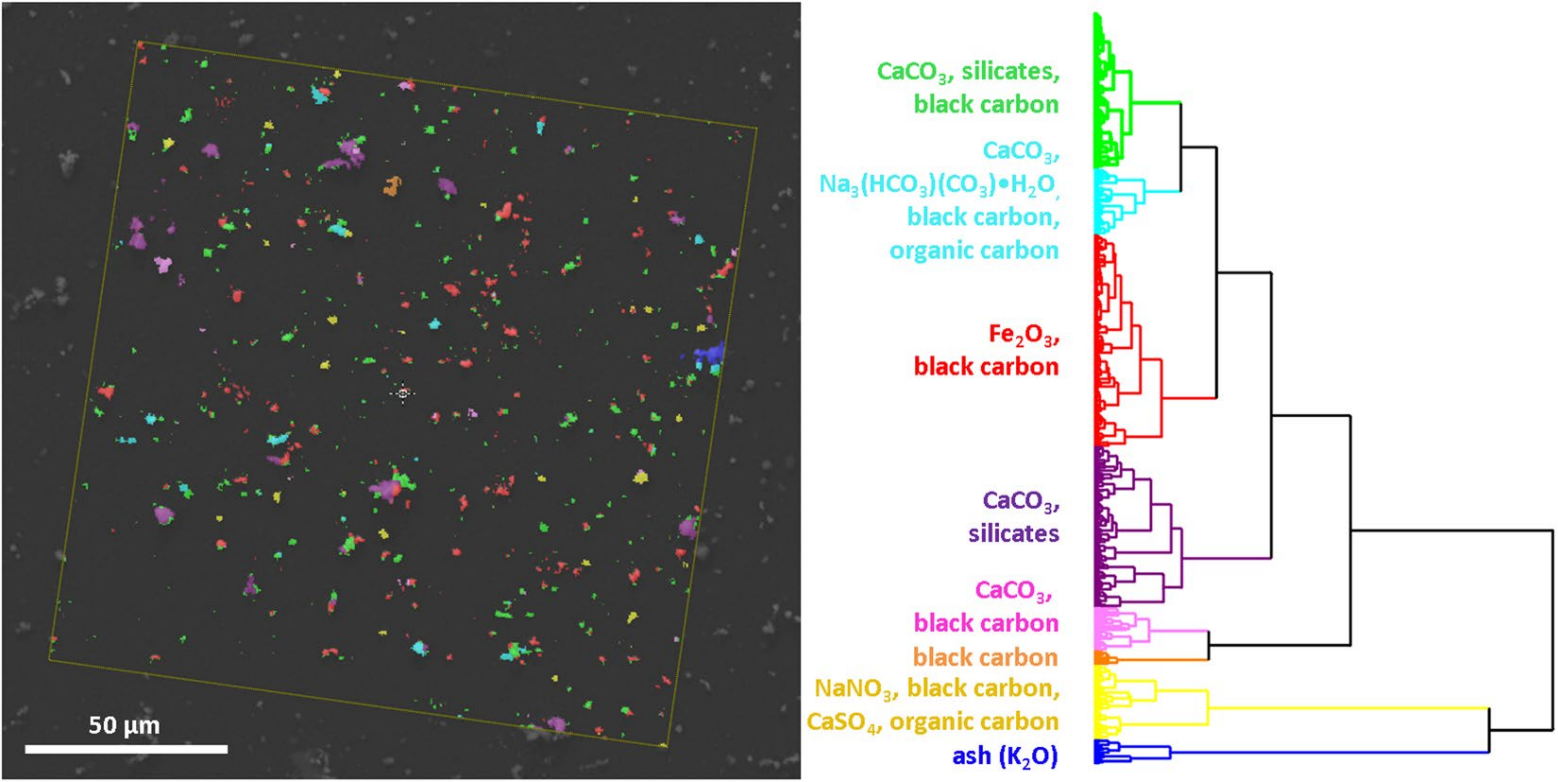
Superposition of the SEM image of the aerosol sample with the virtual image of the HCA of the MSHSI dataset and assignment of chemical species to the sub-clusters, based on extracted cluster spectra.

The analysis of the HCA of the MSHSI dataset revealed at least eight sub-clusters, which can be assigned to different chemical species (Fig. 5). All sub-clusters exhibit strong internal mixing of the single aerosol particles. The yellow sub-cluster shows typical urban aerosol composition and represents a minor part of the overall particle composition. The blue sub-cluster, which is closely connected in terms of inter-cluster distance, can also be attributed to the urban background aerosol. The remaining sub-clusters can be attributed to the tearing down of the burned building.

**Figure 6:**
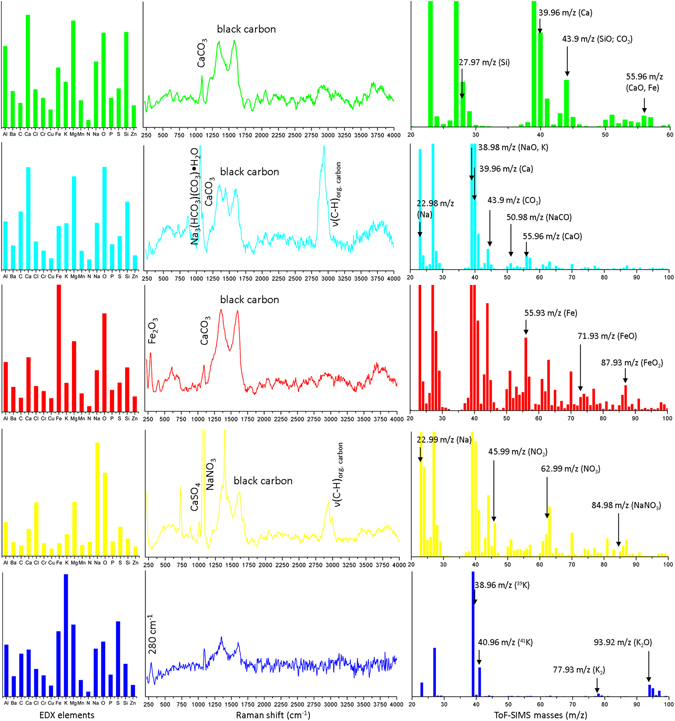
Examples of extracted sub-cluster spectra from the HCA of the environmental aerosol sample and assignment of spectral features.

The assignment of the individual sub-clusters (Fig. 5) to chemical species is achieved by analysing the sub-cluster spectra in parallel (Fig. 6).

The green sub-cluster is dominated by the EDX elements Ca, Mg, O, and Si with contributions from Fe and Na. The RMS dataset exhibits a band at about 1085 cm^−1^ that can be assigned to calcite or aragonite (CaCO_3_) through comparison with reference spectra^26, 37^. Additionally, the Raman spectrum includes a contribution from black carbon (soot)^38^, which can be attributed to the fire at the demolished building. The SIMS spectrum supports this interpretation by exhibiting contributions from the masses (*m*/*z*) 27.97 (Si), 39.96 (Ca), 43.9 (SiO or CO_2_, as a fragment of the carbonate), and 55.96 (Fe or CaO). The green sub-cluster thus exhibits features typical of the building demolition process. The silicates and the CaCO_3_ can be attributed to concrete.

The cyan blue sub-cluster exhibits features in the EDX spectrum similar to those of the green sub-cluster, with an enhanced contribution from carbon. However, the Raman spectrum is supplemented by a dominant band in the spectral region of ν(C–H) stretching vibrations, which indicates the presence of organic carbon, and a sharp band at 1065 cm^−1^. Additionally, the presence of black carbon is indicated. Analysing the SIMS spectrum showed the presence of 22.98 (Na), 38.98 (NaO, K), 39.96 (Ca), 43.9 (CO_2_ from carbonates), 50.98 (NaCO), and 55.96 (CaO, Fe) *m*/*z*. Hence, the presence of some form of sodium carbonate is likely. Comparison of the Raman band at 1065 cm^−1^ with the RRUFF database^26^ shows that it can likely be attributed to trona, a water-bearing sodium hydrogen carbonate with the molecular formula Na_3_(HCO_3_)(CO_3_)·H_2_O that is used as a fire-extinguishing agent. This sub-cluster can thus be attributed to demolition waste and the fire-extinguishing agent.

The EDX spectrum of the red sub-cluster is dominated by Fe and O. The Raman spectrum exhibits a band at 290 cm^−1^ along with bands of CaCO_3_ and black carbon. In addition to the SIMS masses identified in the green sub-cluster, 55.93 (Fe), 71.93 (FeO), and 87.93 (FeO_2_) *m*/*z* are also visible. Comparison with the RRUFF database^26^ of the Raman spectrum and correlating the three MSHSI spectra of the red sub-cluster indicate that this sub-cluster exhibits contributions from hematite, a fingerprint of the structural steel and the technical building equipment.

The purple, pink, and orange sub-clusters are also related to building demolition debris. Therefore, their analysis is not described or shown in detail in Fig. 6. Still, the decreasing inter-cluster distance to the yellow and blue sub-clusters indicate that mineral dust also forms part of the urban background aerosol.

The remaining sub-clusters (yellow and blue) are significantly separated from the other clusters, indicating a different chemical background.

The EDX part of the yellow sub-cluster reveals the presence of Cl, Na, and O, with contributions of S. The Raman spectrum is dominated by the three distinct Raman bands of NaNO_3_ at 725, 1070, and 1390 cm^−1^, as well as the contributions from black carbon and organic carbon (ν(C–H)). The presence of NaNO_3_ is also confirmed by the presence of 22.99 (Na), 45.99 (NO_2_), 63.99 (NO_3_), and 84.98 (NaNO_3_) *m*/*z* in the SIMS spectrum. Additionally, the presence of NaCl, which is not visible in the RMS and SIMS datasets, can be extracted from the EDX dataset. The presence of CaSO_4_ is indicated by the Raman band at 1017 cm^−1^ and the sulphur signal from the EDX dataset. This sub-cluster represents the expected typical aerosol composition from the city of Vienna based on the season.

The blue sub-cluster is characterized by high potassium content in the EDX spectrum and the SIMS spectrum, indicated by the masses 38.96 (^39^K) and 40.96 (^41^K) *m*/*z*. The SIMS spectrum also displays the masses 77.93 *m*/*z* (K_2_) and 93.92 *m*/*z* (K_2_O). The Raman spectrum exhibits a high noise level. However, the contribution from black carbon and the Raman band of CaCO_3_ are still visible. Additionally a band at about 280 cm^−1^ is present, which can be assigned to K–O containing species^26^. Hence, the blue sub-cluster can be attributed to ash, pointing to the influence of wood combustion, which is known to be present in Vienna.

The application of HCA to the MSHSI dataset of the environmental aerosol sample assisted the identification of single precipitated particles and their attribution to different sources. The different contributions from the demolition of a building were clarified by analysing the combined EDX, Raman, and SIMS spectra. Several of the species were not visible in all three spectra, such as the silicates, K_2_O, or the organic carbon. Therefore, the analysis of this dataset in parallel improves the analytical interpretation and enables a detailed understanding of the sample composition. However, some species, such as CaCO_3_ and Fe_2_O_3_, could only be identified by analysing at least two or even all three of the spectra.

## Discussion

The decisive advantages of image-based CDS by multivariate MSHSI are demonstrated via the combined analysis of copper sulphide particles. EDX, RMS, and SIMS SPDCs unravelled the chemical composition of these particles by merging the CuS signals from each of the three different methods within the same sub-cluster of the PCA-HCA dendrogram. The combinational analysis of the complementary imaging techniques was also verified via the subsequent k-means clustering.

Additional benefits of this method are shown by the three subsequent practical examples. For the cell sample, the spatial allocation of the bromine-containing drug (EDX and SIMS) as well as the correlation of the SIMS fragments of lipids with the –CH_2_ band of the RMS could be extracted from the complex MSHSI dataset. In the case of the technical ceramic sample, the 3Y-ZrO_2_ could be identified by all three analytical methods, while the chemical information about the mullite matrix (3Al_2_O_3_·2SiO_2_) was accessible by only EDX and SIMS. The presence of diamond and ZrSiO_4_ could only be extracted from the RMS dataset by VCA. Chemical analysis of the environmental aerosol sample required the combined interpretation of the cluster spectra from all three techniques: silicates (EDX), black and organic carbon (EDX and RMS), ash (EDX and SIMS), sodium nitrate (EDX, RMS, and SIMS), and sodium hydrogen carbonate (RMS and SIMS).

Multivariate analysis of a fused multisensor hyperspectral imaging dataset, obtained from complementary chemical imaging techniques, provides a unique method of image-based chemical structure determination, where the linkage of analytical information across various individual techniques is proven by the combined statistical approach. Hence, joint clustering of different elemental, vibrational, and mass spectrometric features allows direct interpretation of the investigated chemical compound or structure. Applying and combining complementary techniques of comparable spatial resolutions minimizes the risk of overlooking analytical information. Furthermore, cross-correlating different methods helps to prevent the over-interpretation of insufficient data. Hence, MSHSI provides a more focused method of image-based chemical structure determination. By applying three-dimensional or temporal-correlated imaging techniques, multivariate analysis of fused higher-dimensional or dynamical multisensor hyperspectral imaging datasets will deepen the understanding of complex materials and processes.

## Methods

### Materials, sampling, and substrates

For imaging of CuS and atmospheric particles, aluminium targets, obtained by sputtering highly purified aluminium onto microscope cover slips^39^, were used as substrates.

CuS particles were purchased from Alfa-Aesar (99.8%, Alfa-Aesar, Ward Hill, MA, USA, CAS No. 1317-40-4). The liquid suspension consisting of the CuS particles and highly purified water (Milli-Q Millipore System) was nebulized using an aerosol generator (TOPAS aerosol generator ATM 220). A Sioutas cascade impactor with a Leland Legacy pump (both SKC, PA, USA) was deployed at a flow rate of 9 L min^−1^ to precipitate the nebulized CuS particles onto the aluminium targets. The whole procedure was carried out so as to ensure a suitable spatial distribution of the CuS particles on the aluminium surface.

For the imaging of the cell samples, P31 tumour cells were grown in RPMI medium with 10% FBS and 1% penicillin/streptomycin on gold-coated silicon wafers (~24 h). Then the cells were treated with evofosfamide (50 µM). The cells were rinsed with deionised water and dried under vacuum conditions.

The ceramics sample was synthesized as described by Konegger *et al*.^31, 32^. A polished cross-section of the sample was analysed using the spectroscopic techniques without any further processing.

The aerosol sample was taken on 20 November 2016 at the Getreidemarkt in Vienna at the TU Wien. The sampling onto the described aluminium-coated glass cover slips was also done using a Sioutas cascade impactor in combination with a Leland Legacy pump at a flow rate of 9 L min^−1^. Sampling time was 1.5 h, resulting in a total sampled air volume of 810 L. For the combined imaging, particles sampled on the second stage, with an aerodynamic particle diameter cut-off between 2.5 and 1 µm, were chosen.

### Raman micro-spectroscopic imaging

Raman imaging was performed using a WITec alpha 300RSA + Raman microscope. This instrument is equipped with four Raman lasers (a 488 nm DPSS, a 532 nm frequency-doubled NdYAG, a 632.8 nm HeNe, and a 785 nm diode laser) and two lens-based spectrographs. The spectrograph used for the UV/VIS spectral region is equipped with a 300, 600, and 1200 lines per mm grating and an Andor Newton electron multiplying charge-coupled device (EMCCD). The spectrograph used for the NIR spectral region is equipped with a 300 and 600 lines per mm grating and an Andor iDus Deep Depletion CCD. Raman images were recorded using the software package WITec Control 4.1. The parameters for Raman imaging of the four samples are summarized in Table 1.

**Table 1:** Experimental parameters for Raman imaging of the different samples.

	CuS particles	Cell samples	Ceramics	Aerosol Sample
Laser & power	488 nm, 230 µW	632.8 nm, 6 mW	488 nm, 3 mW	488 nm, 1.1 mW
grating	1200 lines mm^−1^	300 lines mm^−1^	1200 lines mm^−1^	600 lines mm^−1^
CCD mode	EMCCD	conventional	EMCCD	EMCCD
EMCCD gain	230		230	230
objective & NA	100×, 0.9	100×, 0.9	50×, 0.8	100×, 0.9
integration time (s)	0.07	0.7	0.02	0.07
imaging area (µm)	100 × 100	80 × 50	200 × 200	200 × 200
sampled pixels	400 × 400	400 × 250	800 × 800	800 × 800
nominal spatial resolution	250 nm	200 nm	250 nm	250 nm
confocal spatial resolution	200 nm	260 nm	225 nm	200 nm

### Electron microscopy and energy-dispersive X-ray imaging

Electron microscopy and EDX imaging was applied using a FEI Quanta 200 electron microscope with an EDAX EDX detector. Scanning electron microscope images were obtained in secondary and backscattered electron mode. EDX imaging was done at a resolution of 1024 × 768 pixels and an acceleration voltage of 20 kV. EDX images were accumulated for about one hour to achieve reasonable signal-to-noise ratios of the elemental distributions. The parameters for EDX imaging of the four samples are summarized in Table 2.

**Table 2:** Experimental parameters for SEM-EDX imaging of the different samples.

	CuS particles	Cell samples	Ceramics	Aerosol Sample
Magnification	2600×	4000×	1500×	1200×
SEM acceleration voltage (kV)	10	5	25	10
EDX imaged area (µm)	129 × 99	87 × 67	225 × 173	282 × 216
EDX spatial resolution (nm)	126	85	220	275
Exported elements from EDX	Al, Cu, O, S	Au, Br, C, Ca, Cl, K, Mg, N, Na, O, P, Pd, S	Al, C, Fe, Hf, O, Si, Y, Zr	Al, Ba, C, Ca, Cl, Cr, Cu, Fe, K, Mg, Mn, N, Na, O, P, S, Si, Zn

### ToF-SIMS imaging

ToF-SIMS imaging was performed using a ToF-SIMS^5^ (ION-TOF GmbH, Münster, Germany) equipped with a 25 keV bismuth liquid metal ion gun (LMIG). Burst alignment (BA) mode was applied on all samples with a pulse width of 57.4 ns for the analysis of areas from 100 µm × 100 μm to 300 µm × 300 µm utilizing Bi_3_^+^ primary ion clusters. The primary ion beam was scanned over the region of interest with a repetition rate of 14,300 Hz. Target currents of approximately 0.04 pA and spot size of approximately 200 nm are achieved, but the mass resolution is decreased to *m*/Δ*m* ~ 300. For sputtering, a dual source column (DSC) was operated at 1 keV. In positive ion mode, sputtering was done with O_2_^+^ at a current of 190 nA, and in negative ion mode, sputtering was done with Cs^+^ at a current of 75 nA. All samples were recorded in the non-interlaced sputter mode (the sputtering cycle and the analysis cycle are decoupled and separated by a specified pause time). During the pause time, a low energy electron flood gun (20 eV) was used to compensate surface charging.

### Data preprocessing and handling

The software package WITec Project 4.1 was used for post-processing of the Raman images. Raman spectral images were exported as graphical ASCII files. EDX elemental images were post-processed using the EDAX software package TEAM and exported as CSV files. For data interpretation of the ToF-SIMS images, only masses above a certain threshold intensity were taken into consideration (e.g., 1% of the base peak). Data evaluation was carried out by using ION-TOF SurfaceLab (Version 6.5, ION-TOF GmbH, Münster, Germany). The mass spectra were internally calibrated using well-known and easily assignable masses (e.g., Na^+^, Al^+^, K^+^, C^−^, C_2_^−^, P^−^, PO^−^, etc.). The error in calibration was kept below 100 ppm. The individual mass deviation of signals not used for calibration might be larger. An online mass shift correction and a spatial shift correction within the software package were used to achieve optimum mass assignment as well as highly resolved secondary ion images. For further data treatment, the secondary ion images were exported as BIF6 files.

Single imaging datasets were combined into multisensor hyperspectral datasets using the software package Imagelab (Epina GmbH, Austria). All datasets were imported and aligned to a common background image for each of the four samples. Finally, the individual hyperspectral datasets were combined to give a multisensor hyperspectral datacube as described by Lohninger and Ofner^17^.

The final spatial resolution of the fused datasets is depending on the spatial resolutions of every single method and on the lateral offset as well as the rotation of the single datasets to each other. In general, a maximum decrease by the square-root of 2 of the worst spatial resolution can be expected (at maximum rotation). However, by ensuring a minimized rotational misalignment, spatial resolutions of the fused datasets can be expected to be hardly worse compared to the worst resolution of a single technique (CuS particles: 250 nm; Tumour cells: 260 nm; Ceramic Composite: 225 nm; Environmental aerosol: 275 nm).

SPDCs for the individual elements from the EDX and masses from the SIMS data cubes were defined by applying single intensity descriptors of the signals. For the RMS data cube, triangle template peaks, baseline-corrected and –uncorrected integral descriptors of selected bands as well as centroid descriptors (for the ν(C-H) spectral region) and ratio descriptors of two bands (ZrO_2_-bands in the ceramics dataset) were defined. Further details on the definition of spectral descriptors, spectral preprocessing, and standardization and application of multivariate statistics to the multisensor hyperspectral datasets, as well as introductions to the different statistical methods, can be found in preceding studies^17, 18^.

The authors provide the multisensor data sets and the spectral descriptors used in this study. Further information can be found in the ImageLab data repository (http://www.imagelab.at/en_data_repository.html datasets DS009 - DS012).
